# Comparison of the optical quality after SMILE and FS-LASIK for high myopia by OQAS and iTrace analyzer: a one-year retrospective study

**DOI:** 10.1186/s12886-021-02048-5

**Published:** 2021-08-02

**Authors:** Yewei Yin, Ying Lu, Aiqun Xiang, Yanyan Fu, Yang Zhao, Yuanjun Li, Tu Hu, Kaixuan Du, Shengfa Hu, Qiuman Fu, Xiaoying Wu, Dan Wen

**Affiliations:** 1 Eye Center of Xiangya Hospital, Central South University, Changsha, 410008 Hunan China; 2 Hunan Key Laboratory of Ophthalmology, Changsha, 410008 Hunan China

**Keywords:** SMILE, FS-LASIK, High myopia, OQAS, iTrace, Optical quality

## Abstract

**Background:**

To compare the correction effect and optical quality after small-incision lenticule extraction (SMILE) and femtosecond laser assisted laser in situ keratomileusis (FS-LASIK) for high myopia.

**Methods:**

51 high myopia eyes after SMILE and 49 high myopia eyes after FS-LASIK were enrolled and divided into two groups retrospectively. The OQAS and iTrace analyzer were used for optical quality inspection. Between the two groups the spherical equivalent (SE), astigmatism, uncorrected distant visual acuity (UDVA), strehl ratio (SR), modulation transfer function cutoff frequency (MTF cutoff), objective scatter index (OSI) and wavefront aberrations were analyzed and compared before surgery and at 1, 6 and 12 months after surgery.

**Results:**

After the operation: (1) SE and astigmatism declined and UDVA increased significantly in both groups, and UDVA was better after SMILE than FS-LASIK. (2) SR and MTF cutoff reduced and OSI increased significantly after SMILE and FS-LASIK. SR and MTF cutoff were significantly higher after SMILE than FS-LASIK. OSI was significantly lower after SMILE than FS-LASIK. (3) The total wavefront aberration, total low-order wavefront aberration, defocus and astigmatism aberration as well as trefoil aberration reduced significantly in both groups. The total high-order wavefront aberration increased significantly after FS-LASIK. The spherical and coma aberration increased significantly in both groups. The total high-order wavefront aberration and coma aberration at 1 month were higher after FS-LASIK than SMILE.

**Conclusion:**

The optical quality descended after SMILE and FS-LASIK. SMILE was superior to FS-LASIK at the correction effect and optical quality for high myopia. The combination of OQAS and iTrace analyzer is a valuable complementary measurement in evaluating the optical quality after the refractive surgery.

**Trial registration:**

This is a retrospective study. This research was approved by the ethics committee of Xiangya Hospital and the IRB approval number is 201612074.

## Background

Being a common eye ametropia disease, high myopia has now widely existed in the general population and its incidence rate still keeps increasing so far. Meanwhile, the refractive surgery develops quite quickly in recent years and increasing numbers of patients with high myopia choose the surgery to correct the refractive errors [1, 2]. In the field of corneal refractive surgery, both small-incision lenticule extraction (SMILE) and femtosecond laser assisted laser in situ keratomileusis (FS-LASIK) have been extensively applied [3, 4]. When performing the SMILE surgery, a corneal stromal lenticule is firstly made with the femtosecond laser and then extracted from a small side incision [5]. As a comparison, femtosecond laser is utilized in the FS-LASIK surgery to generate the corneal flap, and excimer laser is applied to ablate the corneal stroma subsequently [6].

In order to effectively evaluate the effect after the refractive surgery, different kinds of examination methods and evaluating indicators were used in previous work [7–9]. Thereinto, the optical quality is believed to be the most important indicator [10]. According to most of clinical data, the optical quality after SMILE is generally better than that after FS-LASIK [11–13]. However, most of these data are obtained from the patients with low and moderate myopia. Due to the high refractive error and limited corneal thickness, the corneal refractive surgery could not be widely performed for the patients with high myopia. Few observations about the optical quality after SMILE and FS-LASIK were done for the high myopic patients. Therefore, it is meaningful to evaluate the optical quality objectively in high myopia correction after SMILE and FS-LASIK.

A comprehensive measurement of multiple indicators is needed for the effective evaluation of the postoperative optical quality. Considering the existing visual quality inspection equipments, both the Optical Quality Analysis System (OQAS, Visiometrics, Terrassa, Spain) and Tracey-iTrace Visual Function Analyzer (iTrace, Texas, USA) could offer an effective and valid method to evaluate the effectiveness and outcomes of corneal refractive surgery [14–16]. In this work, we intended to obtain the objective data of the optical quality for the patients with high myopia and compare the optical quality after SMILE and FS-LASIK by simultaneously applying the OQAS and iTrace analyzer.

## Patients and methods

### Patients and study design

In this retrospective study, the clinical data of the right eyes of 100 patients with high myopia were collected and analyzed. Thereinto, 51 eyes (27 females, 24 males) had the SMILE surgery (hereinafter called SMILE group) and 49 eyes (26 females, 23 males) had FS-LASIK (hereinafter called FS-LASIK group) after considering comprehensively the corneal thickness, refractive errors to be corrected and the personal requirements. The operation date and follow-up for these patients was from November 1st, 2017 to November 31st, 2020 in the Eye Center of Xiangya Hospital, Central South University, China. The inclusion criteria of enrolling these patients was as follows: (1) the age was 18 years old or older, (2) the spherical refraction was from − 6.00 D to − 10.00 D and the astigmatism was from 0 D to − 2.50 D, (3) there was no history of other eye diseases that might impair the patients’ vision, (4) the follow-up duration was more than one year with regular check at 1, 6 and 12 months postoperatively, (5) both the SMILE and FS-LASIK surgery were performed by the same experienced surgeon and the examinations before and after operation were performed by the same experienced technicians.

### Surgical procedures

For the SMILE surgery, the VisuMax 500 kHz femtosecond laser (Carl Zeiss Meditec AG, Germany) was applied to create the corneal stromal lenticule and side incision. The laser energy was 130nJ, and the spacing between the points and rows was both 4.5 μm. The diameter of the lenticules (i.e. programmed optical zone) was 6.0 ~ 6.8 mm, and the corneal cap thickness was 100 ~ 120 μm. The side-cut angle was 90° with a circumferential width of 4.0 mm.

As to the FS-LASIK surgery, the corneal flap was fabricated with VisuMax 500 kHz femtosecond laser with energy of 185nJ. The thickness of the corneal flap was 90 μm, and the diameter of the flap was 8.0 mm with standard 90° hinge and 90° side-cut angle. The ablation on the corneal stroma was accomplished with the excimer laser (VISX Star S4 Custom VSS Excimer Laser System, America) with a repetition rate of 250 Hz.

In the SMILE and FS-LASIK surgery the treatment center was the corneal vertex and no particular adjustments were performed to the manufacturer’s nomograms. Postoperatively, the treatment regimen was identical for both groups, including tobramycin dexamethasone (Alcon Couvreur, Belgium) four times daily in the first week, 0.3% tobramycin (Alcon Couvreur, Belgium) and 0.1% fluorometholone (Santen, Japan) four times daily in the second week, 0.1% fluorometholone (Santen, Japan) and pranoprofen (Senju, Japan) four times daily in the third week, and pranoprofen (Senju, Japan) four times daily in the fourth week, and 0.1% hyaluronic acid sodium (Ursapharm Arzneimittel GmbH, Germany) four times daily in a month.

### Examinations

The examinations were performed before surgery and at 1, 6, 12 months after surgery, including the slit-lamp examination, the eyesight test using the standard logarithmic visual acuity chart and snellen visual acuity chart, the manifest refraction, the OQAS examination and iTrace analyzer examination. The OQAS was applied with 4.0 mm pupil diameter to get the parameters including strehl ratio (SR), modulation transfer function cutoff frequency (MTF cutoff, c/deg) and objective scatter index (OSI). The iTrace analyzer was used with 4.0 mm pupil diameter to get the data of the total ocular wavefront aberrations, including the total wavefront aberration (TWA), total low-order wavefront aberration (TLOA), total high-order wavefront aberration (THOA), defocus aberration (DA), astigmatism aberration (AA), spherical aberration (SA), coma aberration (CA) and trefoil aberration (TA). The examinations were completed by the same technicians respectively.

### Statistical analyses

The SPSS statistical package (Version 25.0; IBM SPSS Inc., Chicago, Illinois, USA) and the Microsoft Excel Software were applied for the statistical analysis. The one-way repetitive measurement and analysis of variance was applied to analyze the difference of spherical equivalent (SE), astigmatism, uncorrected distant visual acuity (UDVA), SR, MTF cutoff, OSI and wavefront aberrations before surgery and at 1, 6 and 12 months after surgery when the data followed the law of normal distribution. Otherwise, the Friedman test was used. The paired t-test was used to analyze the difference of age, SE, astigmatism, UDVA, SR, MTF cutoff, OSI and wavefront aberrations at each point of time between the two groups. And the multiple-factor repetitive measurement and analysis of variance was used to compare the pre and post-operative variation of SE, astigmatism, UDVA, SR, MTF cutoff, OSI and wavefront aberrations between the SMILE and FS-LASIK group. *p* < 0.05 was taken as being statistically significant, and the adjusted alpha i.e., alpha/no. of test, was adopted for the analysis with repeated measurement.

## Results

### Descriptive statistics of the patients, UDVA and the other refractive outcomes

No intraoperative or postoperative complications, such as corneal infection, haze, corneal epithelial ingrowth and elevated intraocular pressure, occurred in both the SMILE and FS-LASIK group. As summarized in Table 1, there were no significant differences between the two groups in terms of age, preoperative and postoperative SE and astigmatism. Both SE and astigmatism significantly decreased after SMILE and FS-LASIK (*p* < 0.05). Between the two groups there were no significant differences in SE and astigmatism at each point of time, and no significant differences in SE and astigmatism’s variation before and after the operation were found (*p*^SE^ = 0.119, *p*^astig^ = 0.782). The preoperative UDVA had no significant difference between the two groups and increased significantly after operation until the 12th month in both groups (*p* < 0.05). The pre and post-operative variation of UDVA had no significant difference between the two groups (*p* = 0.481), and the postoperative UDVAs in the SMILE group were better than those in the FS-LASIK group with significant differences (*p* < 0.05).

**Table 1:** The preoperative and postoperative demographics (mean ± SD) for the SMILE and FS-LASIK group

Parameters		SMILE group	FS-LASIK group	*p*_*1*_-value
Age (years old)		23.9 ± 4.8	24.4 ± 4.9	0.605
SE(D)	Preop	−7.964 ± 0.943	−8.390 ± 1.372	0.078
	Post 1mo	−0.194 ± 0.549*	−0.230 ± 0.487*	0.740
	Post 6mo	−0.156 ± 0.486*	−0.194 ± 0.459*	0.677
	Post 12mo	−0.204 ± 0.461*	−0.334 ± 0.441*	0.169
	*p*_0_-value	0.119		
Astigmatism (D)	Preop	−0.786 ± 0.508	−0.806 ± 0.736	0.846
	Post 1mo	0.005 ± 0.377*	−0.031 ± 0.453*	0.655
	Post 6mo	−0.163 ± 0.321*	−0.102 ± 0.398*	0.326
	Post 12mo	−0.046 ± 0.356*	−0.031 ± 0.352*	0.832
	*p*_0_-value	0.782		
UDVA (LogMAR)	Preop	−1.292 ± 0.274	−1.335 ± 0.289	0.427
	Post 1mo	0.129 ± 0.167*	0.025 ± 0.111*	<0.001
	Post 6mo	0.104 ± 0.110*	0.045 ± 0.121*	0.023
	Post 12mo	0.099 ± 0.106*	0.032 ± 0.176*	0.021
	*p*_0_-value	0.481		

SD: standard deviation. Preop: preoperative. Post 1mo: at 1 month postoperatively. Post 6mo: at 6 months postoperatively. Post 12mo: at 12 months postoperatively. *p*_0_ = *p* value of the difference for the pre and post-operative variation of SE, astigmatism and UDVA between the SMILE and FS-LASIK group. *p*_1_ = *p* value of the difference in age, SE, astigmatism and UDVA at each point of time between the SMILE and FS-LASIK group. When *p* < 0.05, the difference was statistically significant. *Compared with that before the operation, *p* < adjusted alpha

**Fig. 1:**
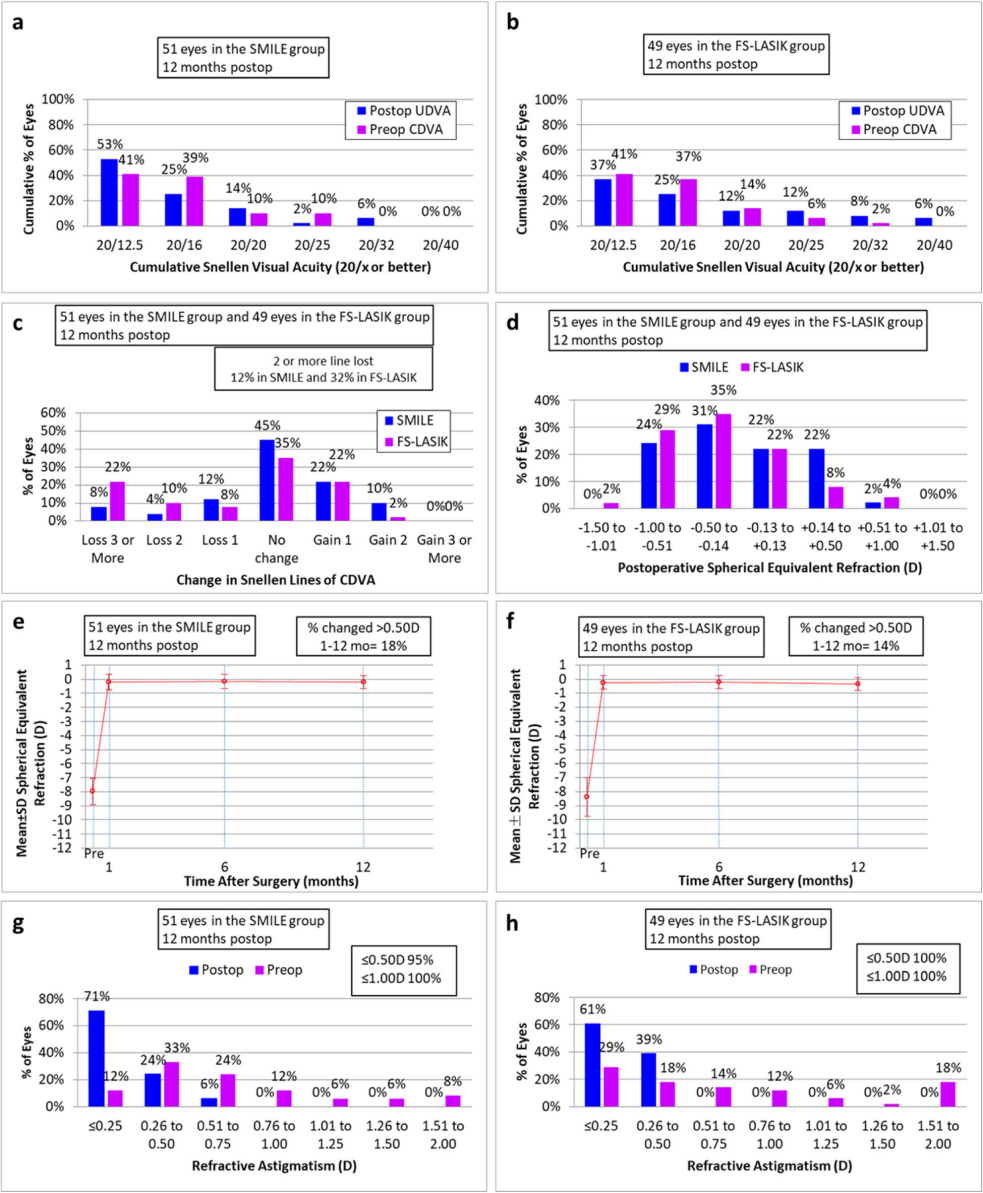
The standard graphs for reporting the visual and refractive outcomes of 51 eyes in the SMILE group and 49 eyes in the FS-LASIK group at 12 months postoperatively

Figure 1 shows the standard graphs for reporting the clinical results of the two corneal refractive surgeries. At 12 months after the operation, 92% (47/51) of treated eyes in the SMILE group and 74% (36/49) in the FS-LASIK group had a postoperative snellen visual acuity of 20/20 or better. And the corrected distant visual acuity (CDVA) of 12% of eyes in the SMILE group and 32% in the FS-LASIK group had 2 or more lines lost at 12 months postoperatively. 75 and 65% of eyes achieved postoperative SE within +/− 0.50 D at 12 months after SMILE and FS-LASIK respectively. From the 1st to the 12th month, 17% of eyes in the SMILE group and 14% in the FS-LASIK group had > 0.50D change of SE. At 12 months postoperatively, 95 and 100% of eyes had the refractive astigmatism less than or equal to +/− 0.50D in the SMILE and FS-LASIK group respectively.

### SR, MTF cutoff and OSI

Preoperatively, there were no significant differences in SR, MTF cutoff and OSI between the SMILE and FS-LASIK group (*p*^SR^ = 0.259, *p*^MTF cutoff^ = 0.151, *p*^OSI^ = 0.719). After the surgery, SR and MTF cutoff in the two groups both reduced significantly (*p* < 0.05) except the MTF cutoff at 12 months in the SMILE group (*p* = 0.055). Compared with those in the FS-LASIK group, the postoperative SR and MTF cutoff were obviously more in the SMILE group at each point of observing time (*p* < 0.05). The pre and post-operative variation of SR and MTF cutoff had no significant difference between the two groups (*p*^SR^ = 0.057, *p*^MTF cutoff^ = 0.062). On the other hand, OSI increased significantly after both surgeries (*p* < 0.05). The pre and post-operative variation of OSI showed significant difference between the two groups (*p* < 0.001). Moreover, after the operation OSI in the FS-LASIK group was much more than that in the SMILE group (*p* < 0.05) as summarized in Table 2.

**Table 2:** The variation of the SR, MTF cutoff and OSI with time in the SMILE and FS-LASIK group (mean ± SD)

	Preop	Post 1mo	Post 6mo	Post 12mo	*p*_0_-value
	SR				
SMILE group	0.260 ± 0.064	0.221 ± 0.069*	0.212 ± 0.057*	0.233 ± 0.069*	0.057
FS-LASIK group	0.245 ± 0.077	0.185 ± 0.062*	0.175 ± 0.062*	0.178 ± 0.047*	
*p*_1_-value	0.259	0.010	0.007	<0.001	
	MTF cutoff				
SMILE group	43.684 ± 7.955	39.161 ± 10.711*	37.547 ± 10.297*	40.552 ± 10.005	0.062
FS-LASIK group	41.462 ± 9.809	32.520 ± 12.305*	32.361 ± 10.764*	31.489 ± 9.626*	
*p*_1_-value	0.151	0.007	0.029	<0.001	
	OSI				
SMILE group	0.49 ± 0.24	0.88 ± 0.53*	0.84 ± 0.44*	0.80 ± 0.53*	<0.001
FS-LASIK group	0.59 ± 0.33	1.63 ± 1.01*	1.39 ± 0.92*	1.29 ± 0.72*	
*p*_1_-value	0.719	<0.001	0.001	0.001	

SD: standard deviation. Preop: preoperative. Post 1mo: at 1 month postoperatively. Post 6mo: at 6 months postoperatively. Post 12mo: at 12 months postoperatively. *p*_*0*_ = *p* value of the difference for the pre and post-operative variation of SR, MTF cutoff and OSI between the SMILE and FS-LASIK group. *p*_1_ = *p* value of the difference in SR, MTF cutoff and OSI at each point of time between the SMILE and FS-LASIK group. When *p* < 0.05, the difference was statistically significant. * Compared with that before the surgery, *p* < adjusted alpha

### The wavefront aberrations

As shown in Table 3, no significant differences in TWA, TLOA and THOA before the operation were found between the SMILE and FS-LASIK group. After the operation, both TWA and TLOA reduced significantly in the two groups (*p* < 0.05), and their pre and post-operative variation showed no significant difference between the two groups (*p*^TWA^ = 0.115, *p*^TLOA^ = 0.061). There were no significant differences in postoperative TWAs and TLOAs at each point of time between the two groups. The THOAs did not change significantly after SMILE. In contrast, THOA increased significantly after FS-LASIK (*p* < 0.05) and was significantly more than that in the SMILE group at 1 month (*p* = 0.033). The pre and post-operative variation of THOA showed no significant difference between the two groups (*p* = 0.115).

**Table 3:** The variation of total wavefront aberrations with time in the SMILE and FS-LASIK group (mean ± SD)

	Preop	Post 1mo	Post 6mo	Post 12mo	*p*_0_-value
	TWA				
SMILE group	7.896 ± 2.842	1.910 ± 1.830*	2.014 ± 1.665*	2.156 ± 1.791*	0.115
FS-LASIK group	8.797 ± 3.459	1.965 ± 1.582*	1.766 ± 1.509*	2.376 ± 1.761*	
*p*_1_-value	0.122	0.841	0.440	0.539	
	TLOA				
SMILE group	7.756 ± 2.967	1.812 ± 1.778*	1.900 ± 1.585*	2.052 ± 1.687*	0.061
FS-LASIK group	8.780 ± 3.446	1.717 ± 1.476*	1.572 ± 1.444*	2.226 ± 1.683*	
*p*_1_-value	0.099	0.783	0.293	0.623	
	THOA				
SMILE group	0.565 ± 0.775	0.543 ± 0.512	0.594 ± 0.597	0.598 ± 0.665	0.115
FS-LASIK group	0.485 ± 0.384	0.789 ± 0.727*	0.714 ± 0.575*	0.741 ± 0.642*	
*p*_1_-value	0.514	0.033	0.307	0.251	

SD: standard deviation. Preop: preoperative. Post 1mo: at 1 month postoperatively. Post 6mo: at 6 months postoperatively. Post 12mo: at 12 months postoperatively. TWA: total wavefront aberration. TLOA: total low-order wavefront aberration. THOA: total high-order wavefront aberration. *p*_*0*_ = *p* value of the difference for the pre and post-operative variation of TWA, TLOA and THOA between the SMILE and FS-LASIK group. *p*_1_ = *p* value of the difference in TWA, TLOA and THOA at each point of time between the SMILE and FS-LASIK group. When *p* < 0.05, the difference was statistically significant. * Compared with that before the surgery, *p* < adjusted alpha

The defocus aberration (DA) and astigmatism aberration (AA) both decreased significantly at 1, 6 and 12 months after the two surgeries (*p* < 0.05). No significant differences for their pre and post-operative variations were found between the SMILE and FS-LASIK group (*p*^DA^ = 0.085, *p*^AA^ = 0.119). Moreover, the DAs and AAs had no significant difference between the two groups, preoperatively and postoperatively. The spherical aberration (SA) and coma aberration (CA) increased significantly at 1, 6 and 12 months after the two surgeries and their pre and post-operative variation showed no significant difference between the two groups (*p*^SA^ = 0.467, *p*^CA^ = 0.216). In addition, there were no significant differences in SAs and CAs between the two groups preoperatively and postoperatively except CA at 1 month which was more in FS-LASIK (*p* = 0.019). Furthermore, the trefoil aberration (TA) reduced significantly after the operation in both groups (*p* < 0.05) and its pre and post-operative variation had no significant difference between the two groups. It’s also illustrated that there were no significant differences in TAs between the two groups preoperatively and postoperatively in Table 4.

**Table 4:** The variation of the wavefront aberrations with time in the SMILE and FS-LASIK group (mean ± SD)

	Preop	Post 1mo	Post 6mo	Post 12mo	*p*_0_-value
	DA				
SMILE group	7.814 ± 2.800	1.714 ± 1.797*	1.824 ± 1.598*	1.972 ± 1.670*	0.085
FS-LASIK group	8.671 ± 3.370	1.651 ± 1.458*	1.470 ± 1.462*	2.152 ± 1.704*	
*p*_1_-value	0.133	0.856	0.250	0.613	
	AA				
SMILE group	0.967 ± 0.557	0.381 ± 0.357*	0.391 ± 0.300*	0.441 ± 0.431*	0.119
FS-LASIK group	1.207 ± 0.988	0.420 ± 0.434*	0.380 ± 0.344*	0.427 ± 0.350*	
*p*_1_-value	0.134	0.513	0.940	0.869	
	SA				
SMILE group	0.081 ± 0.179	0.223 ± 0.374*	0.258 ± 0.452*	0.241 ± 0.487*	0.467
FS-LASIK group	0.053 ± 0.115	0.309 ± 0.536*	0.270 ± 0.400*	0.297 ± 0.388*	
*p*_1_-value	0.338	0.316	0.887	0.503	
	CA				
SMILE group	0.235 ± 0.167	0.367 ± 0.349*	0.396 ± 0.411*	0.407 ± 0.461*	0.216
FS-LASIK group	0.265 ± 0.298	0.571 ± 0.514*	0.537 ± 0.435*	0.574 ± 0.526*	
*p*_1_-value	0.543	0.019	0.124	0.092	
	TA				
SMILE group	0.213 ± 0.152	0.172 ± 0.130*	0.163 ± 0.119*	0.170 ± 0.172*	0.082
FS-LASIK group	0.246 ± 0.200	0.140 ± 0.090*	0.142 ± 0.097*	0.130 ± 0.085*	
*p*_1_-value	0.363	0.162	0.215	0.142	

SD: standard deviation. Preop: preoperative. Post 1mo: at 1 month postoperatively. Post 6mo: at 6 months postoperatively. Post 12mo: at 12 months postoperatively. DA: defocus aberration. AA: astigmatism aberration. SA: spherical aberration. CA: coma aberration. TA: trefoil aberration. *p*_*0*_ = *p* value of the difference for the pre and post-operative variation of the wavefront aberrations between the SMILE and FS-LASIK group. *p*_1_ = *p* value of the difference in the wavefront aberrations at each point of time between the SMILE and FS-LASIK groups. When p < 0.05, the difference was statistically significant. * Compared with that before the surgery, p < adjusted alpha

## Discussion

As the safest myopic surgeries in recent years, SMILE and FS-LASIK are widely applied for myopia correction. The theory and procedure are different between the two surgeries. But similarly, for high myopia correction, more corneal tissue will be removed in the two surgeries and the corneal refractive power is more likely to be instable. However, few studies have focused on the correction effect and optical quality after SMILE and FS-LASIK in high myopia. Hence this study worked on evaluating and comparing the correction effect and optical quality after SMILE and FS-LASIK for patients with high myopia.

The visual acuity and refractive power are the most commonly used indicators to illustrate the effectiveness of myopic surgery. In this work, both SMILE and FS-LASIK could effectively correct the refractive errors and improve the naked visual acuity of the patients with high myopia, similar with the other studies [17–19]. This study also found that the postoperative visual acuity (UDVA) and refractive results (SE and refractive astigmatism) after SMILE were better than those after FS-LASIK. Hence one can see that SMILE had an advantage over FS-LASIK at achieving more satisfactory visual acuity and refractive results, at least for a year. It was noteworthy that a loss of visual acuity occurred at 12 months after SMILE and FS-LASIK, consistent with the change of spherical equivalent refraction. We think the main reason for the loss is that the subjects are all high myopia patients, as it has been proved that the refractive regression is more likely to occur in patients with high myopia [20, 21]. We also analyzed the individual eyes with visual acuity loss and speculated that it might be the result of abnormal accommodation function before surgery in these young high myopia patients [22]. A previous research also concluded that a decrease in amplitude of accommodation and facility of accommodation might result in some of the near-vision problems in younger myopes in early postoperative days after refractive surgery [23]. Nevertheless, as we did not measure the accommodation function of all these subjects preoperatively, there is no hard proof for this assumption.

Due to the postoperative corneal deformation, the increased corneal scatter, wavefront aberrations and other factors might affect the optical quality [24–26]. Hence this study measured and evaluated the postoperative objective optical quality. Because of the limitation of a single detection equipment, this study used OQAS and iTrace analyzer simultaneously which were both widely applied for objective optical quality inspection and whose repeatability had been adequately verified in the previous work [14–16]. Based on the double-pass technique, OQAS is known as the device that can quantitatively provide the data of ocular scatter [27, 28]. The most dominant parameters detected by OQAS include: (1) SR that indicates the convergence ratio of light intensity in the image field of an optical system with aberrations, and the higher the value is, the better the optical quality is; (2) MTF cutoff that characterizes the spatial frequency corresponding to the minimum resolution of human eyes in the modulation transfer function curve, and the higher the value is, the better the optical quality becomes; (3) OSI that objectively reflects the scatter of the refractive medium, and the higher the value is, the muddier the refractive media is. Another evaluation instrument, i.e. iTrace analyzer, is composed of a corneal topographer and an aberrometer whose working principle is ray tracing. It is applicable for the measurement of corneal, internal and total ocular wavefront aberrations [16]. Wavefront aberrations are distortions in the phase of light entering the eye, which leads to the defects in image-forming and thereby decreasing the quality of vision [29, 30]. They could be caused by the non-optimal surface shapes, irregularities and misalignments in the eye’s optical elements. It’s generally accepted that as the wavefront aberrations were corrected, the visual sensitivity, night vision, incidence of glare and halo would significantly improve. They could be mathematically represented as the sum of a series of polynomial functions of different orders, and the higher the wavefront aberration is, the more the visual quality is affected. In particular, the high-order aberrations have a greater impact on the visual quality. This study applied iTrace analyzer to measure the total ocular wavefront aberrations before and after the operation, including TWA, TLOA, THOA, etc.

As indicated by the variation of SR, MTF cutoff and OSI in this study, the optical quality descended constantly for one year after both SMILE and FS-LASIK. This finding was different from some previous observations about SMILE surgery [31–33]. Miao et al. used the OQAS to evaluate the optical quality after SMILE and found that the optical quality was not significantly reduced [31]. Niu et al. got a result that for high myopia correction, MTF cutoff declined slightly at 3 months after SMILE but recovered to the preoperative value at the one-year follow-up, and OSI increased at 20 days after surgery but gradually declined to the pre-operative level at 3 months [32]. Qin et al. find that no significant difference was found in SR or MTF cut-off in SMILE for high myopia correction before surgery or at any time point after surgery [33]. And few reports were found about the optical quality for high myopia after FS-LASIK evaluated by OQAS. The specific reasons for these differences are unknown, but it was worth the attention of refractive surgeons that there is a decline in the optical quality after the operation.

The results about SR, MTF cutoff and OSI also suggested that SMILE achieved better optical quality than FS-LASIK. In especial, the MTF cutoff at 12 months in SMILE returned to the preoperative level without any difference, possibly indicating the slightly recovery of optical quality as time progressed. Similar research by OQAS was rare between SMILE and FS-LASIK. We considered that the superiority of SMILE here was possibly related to the size of optical zone and high pulse frequency of femtosecond laser. The better optical quality was often accompanied by the larger cutting area in the corneal refractive surgery [34]. It’s indicated that SMILE had a comparatively larger effective optical zone than FS-LASIK though a similar optical zone was programmed before the operation [35, 36]. Besides, the high pulse frequency of femtosecond laser in SMILE is able to alleviate the inflammation reaction and corneal damage, and then induces limited potential loss of the corneal transparency and less scatter when compared with FS-LASIK [5, 37]. Hence, from the perspective of SR, MTF cutoff and OSI, this study suggested that it is more reasonable to have SMILE surgery for the population with high optical quality requirements.

The wavefront aberration provides another objective method to evaluate the postoperative optical quality. In this study, the TWA, TLOA, DA, AA, SA, CA and TA showed a similar increasing or decreasing trends after SMILE and FS-LASIK, except the CA at 1 month postoperatively. And the THOA did not change significantly after SMILE while showed an upward trend after FS-LASIK. These results indicated that except the THOAs and CA, SMILE and FS-LASIK had no obvious differences in introducing wavefront aberrations, especially the lower order aberrations. Because the refractive errors were effectively corrected after the two surgeries, the DA and AA decreased obviously. The DA and AA accounted for the majority of the total aberrations, hence the TLOA and TWA also decreased after the operation. As to the higher order aberrations, the increasing trend was similar to the previous studies [12, 38, 39]. Due to the surgery incision and the postoperative wound healing, the asymmetry of eye plane such as irregularity, inclination and decentration might occur, which could be reflected by CAs [24]. Due to the cutting of nerve endings when making the corneal flap in FS-LASIK, the quality of tear film became poor and might also affect the corneal aberrations [40–42]. And a minimal displacement of the corneal flap in FS-LASIK could obviously affect the higher-order aberrations. Besides, SMILE brings less damage to the anterior stromal layer, and the heat load generated by femtosecond laser is lower than that by excimer laser, which both would reduce the influence on central asphericity of cornea [43]. In addition, through the aspheric scanning pattern without scanning interval, SMILE can minimize the higher order aberrations introduced by surgery, while FS-LASIK adopts the excimer laser with scanning interval and would introduce more surgically-induced higher order aberrations [44]. Therefore, there is a disadvantage of FS-LASIK against SMILE in terms of the postoperative higher-order wavefront aberrations. In sum, it proved the advantage of SMILE on the optical quality over FS-LASIK.

Additionally, in this study the change of higher-order aberrations after SMILE or FS-LASIK was not exactly the same with the other researches [24, 45, 46]. It might be related to the different refractive diopters, inspection equipments and assessing parameters, detecting conditions, surgery equipments and procedures, surgeons, etc. For example, by using Sirius System, Jin et al. found that SMILE showed better optical quality than FS-LASIK at larger pupil diameter [13]. Yet no observations of optical quality at different pupil diameters were made in our study. Besides, the results reported in this work cannot represent the general myopic population as the preoperative spherical refraction was from − 6.00 D to − 10.00 D and the astigmatism was from 0 D to − 2.50 D. We expect more studies to focus on the optical quality of patients with high myopia after different corneal refractive surgeries. And further work is needed to prolong the observation time and increase the sample sizes in order to detect the optical quality more deeply of patients with high myopia after SMILE and FS-LASIK.

## Conclusions

The data reported in this work indicated that both SMILE and FS-LASIK could improve the visual acuity of the patients with high myopia. And the optical quality descended after the operation. The better postoperative visual acuity and refractive results, the higher SR and MTF cutoff, the lower OSI together with the different change of higher order aberrations illustrated that SMILE was superior to FS-LASIK at the correction effect and optical quality for high myopia population. In addition, the combined application of OQAS and iTrace analyzer is a valuable complementary measurement in evaluating the optical quality after the refractive surgery.

## Data Availability

The datasets analysed during the current study are available from the corresponding author on reasonable request.

## Abbreviations

SMILE: Small incision lenticule extraction
FS-LASIK: Femtosecond laser-assisted in situ keratomileusis
UDVA: Uncorrected distance visual acuity
CDVA: Corrected distance visual acuity
SE: Spherical equivalent refraction
SR: Strehl ratio
MTF cutoff: Modulation transfer function cutoff frequency
OSI: Objective scatter index
WA: Wavefront aberration
TWA: Total wavefront aberration
TLOA: Total low-order wavefront aberration
THOA: Total high-order wavefront aberration
DA: Defocus aberration
AA: Astigmatism aberration
SA: Spherical aberration
CA: Coma aberration
TA: Trefoil aberration
